# Association of UGT1A1*6 polymorphism with irinotecan-based chemotherapy reaction in colorectal cancer patients: a systematic review and a meta-analysis

**DOI:** 10.1042/BSR20200576

**Published:** 2020-10-21

**Authors:** Xiaoyun Zhu, Ruchao Ma, Xin Ma, Gang Yang

**Affiliations:** 1 Department of Gastroenterology, Gansu Provincial Hospital, Lanzhou, Gansu 730030, China; 2 Department of Cardiology, Lanzhou University Second Hospital, Lanzhou, Gansu 730030, China

**Keywords:** colorectal cancer, irinotecan, meta-analysis, response, rs4148323, UGT1A1

## Abstract

Colorectal cancer (CRC) is a leading cause of cancer-related deaths across the world. Irinotecan (IRI) is commonly used to treat CRC, and IRI-based chemotherapy is linked with adverse reaction and the efficacy of the treatment regimen. The gene *UGT1A1* plays a central role in the IRI metabolic pathway. A polymorphism UGT1A1*6 has been widely researched which may be related to response of IRI-based chemotherapy in CRC. All relevant studies were strictly searched from PubMed, Embase, Cochrane Library and Web of Science databases to explore the associations between UGT1A1*6 and response of IRI-based chemotherapy with CRC. Nine articles comprising 1652 patients were included in the final combination. Meta-analysis showed G allele or GG had a lower risk of severe late-onset diarrhea compared with A/AA in allele model and homozygote model (G vs. A: OR = 0.53, 95% CI: 0.28–0.99, *P*=0.05; GG vs. AA: OR = 0.48, 95% CI: 0.23–0.99, *P*=0.05), no significant association was observed in other models. In addition, a significant association between UGT1A1*6 and neutropenia was observed in all models (G vs. A: OR = 0.57, 95% CI: 0.46–0.71, *P*=0.00; GG vs. AA: OR = 0.28, 95% CI: 0.17–0.45, *P*=0.01; GA vs. AA: OR = 0.42, 95% CI: 0.26–0.70, *P*=0.00; GG+GA vs. AA: OR = 0.32, 95% CI: 0.20–0.52, *P*=0.00; GG vs. AA+GA: OR = 0.40, 95% CI: 0.22–0.71, *P*=0.00), whereas, no relationship was found between UGT1A1*6 and clinical response among the different genotypes. UGT1A1*6 may be considered as a biomarker for IRI-based chemotherapy in CRC.

## Background

Colorectal cancer (CRC) is the third most common cancer and the most common cause of deaths all over the world [1]. Although the colon-endoscopy is extensively used to screen the high-risk patients, and some new biomarkers are widely used, such as carcino-embryonic antigen (CEA) and carbohydrate antigen 199 (CA199) [2], the early diagnosis of CRC is difficult, and some patients reach a critical size to produce complications. Hence, the incidence rate of CRC is obviously higher among exposed populations, and the therapy of CRC is still on the way. Recently, some new oncogenes were found which play a pivotal role, and many new gene-related biomarkers were widely researched [3,4]. Irinotecan (IRI) is a widely used chemotherapeutic drug in malignant cancer especially in CRC, which can prolong survival time and improve the prognosis in CRC patients [5]. However, the responses of the drug present obvious differences in different individuals [5,6], so a plenty of researches have attempted to explore the reasons. Genome-wide association studies have demonstrated that many coding or non-coding variants, especially low-frequency or rare coding variants are related to drug response or adverse effects [7], and the researchers found the value for CRC patients to genotyping in cancer chemotherapy [8].

A large number of studies found UDP-glucuronosyltransferase (UGT) enzymes involved in the metabolism of IRI, which take part in glucuronidation and transform the active metabolite IRI (SN38) into SN38 glucuronide (SN38G) [9]. UGT enzymes are encoded by the UGT gene family [10], which consists of a series of UGT1As. Uridine diphosphate glucoronosyltransferase 1A1 is a member of UGT gene family, which is located in 2q37.1 and has five exons (NG_033238). Previous studies confirmed that UGT1A1 was a key enzyme in glucuronidation, and suggested that UGT1A1 gene polymorphism was closely related to metabolism of IRI [11]. The SNP UGT1A1*6 (rs4148323) is a missense polymorphism, which results in single amino acid change (Gly^71^Arg) of the *UGT1A1* gene (https://www.ncbi.nlm.nih.gov/projects/SNP/snp_ref.cgi?rs=4148323). Extensive studies have researched the response of IRI in CRC patients exposing different genotypes of UGT1A1*6 [12–20], but the conclusion is still controversial. Although a recent meta-analysis performed to analyze the difference in adverse reaction and therapeutic response (TR) between IRI-administered cancer patients with different UGT1A1*6 genotypes [21], and explored the association between cancer and UGT1A1*6, and performed the subgroup analysis; the association between CRC and UGT1A1*6 did not explain deeply. Therefore, we performed a meta-analysis to comprehensively investigate the association between UGT1A1*6 polymorphism and response of IRI-based chemotherapy with CRC.

## Methods

### Search strategy

Eligible studies were obtained from PubMed, Embase, Cochrane Library (Cochrane Center Register of Controlled Trails) and Web of Science databases with the date up to 10 February 2020. The MeSH terms and full-text terms used are as follows: ‘irinotecan’, ‘UGT1A1’, ‘polymorphism’, ‘UGT1A1*6’, ‘rs4148323’, ‘Colorectal Neoplasms’, ‘chemotherapy.’ Furthermore, we perused relevant references to select additional relevant studies.

### Inclusion and exclusion criteria

All eligible studies were selected with the following inclusion criteria: (1) clinical trials and observational studies; (2) studies exploring the association between UGT1A1*6 polymorphism and response of IRI-based chemotherapy with CRC; (3) CRC diagnosis based on pathological examination or confirmed by proctoscope; (4) data were sufficient for tumor response (TR) (including complete response (CR), partial response (PR), stable disease (SD) and progressive disease (PD)), which used the WHO criteria (RECIST, Response Evaluation Criteria in Solid Tumors) [22]; (5) the articles concerning allele frequency which could be sufficient to calculate genotypic odds ratio (OR) with the corresponding 95% confidence intervals (95% CIs) in TR; (6) the toxicity measurements were evaluated on the basis of National Cancer Institute Common Toxicity Criteria for Adverse Events, Grade 3–4 neutropenia and Grade 3–4 diarrhea were considered as severe toxicity [23].

Following studies were excluded: (1) reviews, meta-analyses, case reports, letters, comments or duplicated data; (2) animal experiments; (3) studies with undefined genotypes; (4) studies with no effective data; (4) no criteria of diagnosis were described.

### Data extraction

Two reviewers (X.y.Z. and R.c.M.) independently extracted data using standardized criteria. If they could not form a settled consensus, all the authors must discuss the studies and reach a consensus. Information was carefully extracted as follows in each article: first author’s name, publication year, country, population ethnicity, gender, age, total number of patients, detection genotype methods of UGT1A1*6 polymorphism, the regimen of chemotherapy, IRI dose, response criteria, toxicity criteria and key elements of risk assessment of bias etc.

### Quality assessment

The quality of included articles were assessed according to recommendation of Newcastle–Ottawa scale (NOS) [21]. Eight items were selected for the inclusion of the study, including object selection, comparability among groups and exposure factors. Researches with NOS scores of 0–3, 4–6, 6–9 were considered as low-, medium- and high-quality studies, respectively.

### Statistical analysis

The OR and 95% CI were used to assess UGT1A1*6 polymorphism and response of IRI-based chemotherapy with CRC in Asians. Cochran’s Q test and *I^2^* statistics were employed to evaluate the heterogeneity assumption. If significant heterogeneity existed (*P*<0.05, *I^2^*>50%), the random-effects model will be used to pool ORs. Otherwise, fixed-effects model will be chosen [24]. We evaluated the UGT1A1*6 polymorphism and response of IRI-based chemotherapy with CRC in Asians using five genetic models: allele comparison (G vs. A), homozygote comparison (GG vs. AA), heterozygote comparison (GA vs. AA), dominant comparison (GG+GA vs. AA) and recessive comparison (GG vs. GA+AA). In addition, subgroup analyses were performed in this article based on different countries.

Begg’s regression test and funnel plot used to calculate potential publication bias were tested. Sensitivity analysis was also performed to evaluate the stability of the meta-analysis when the significant heterogeneity existed. All the analyses were performed using the STATA 12.0 software. All statistics were two-tailed and *P*<0.05 was considered as significant.

## Results

### Study characteristics

**Figure 1:**
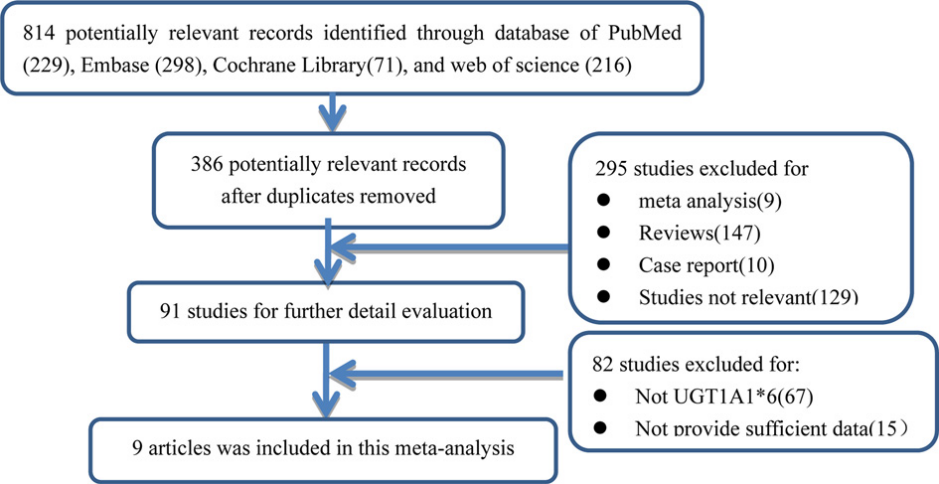
Flow diagram of the study selection process

As shown in Figure 1, 814 potentially eligible records were initially yielded (PubMed: 229, Embase: 298, Cochrane Library: 71, Web of Science: 216). In total, 386 citations were searched after duplicates removal. After different levels of screening based on titles, abstracts and full texts, 156 articles were reviews or meta-analysis, 10 studies were case reports, 129 articles seemed to be not related to this research, 67 studies that were not related to UGT1A1*6 and 15 articles did not provide sufficient data. Nine articles including 1652 patients finally were selected according to the inclusion criteria [12–20]. The clinical characteristics were summarized in Table 1.

**Table 1:** Characteristics and methodological quality of involved studies

Author	Year	Country	Number of patients	Age	Gender (M/F)	Genotyping	Regimen	IRI dose (mg/m^2^)	Response criteria	Toxicity criteria	NOS
Liu	2017	China	661	U	406/255	Sequencing	FLIRI, FOLFOXIRI	150 or 180/U	RECIST	N4	7
Xu	2016	China	69	U	46/23	Sequencing	FOLFIRI, THFC + 5FU	150/3 weeks	RECIST	N3	7
			114	U	78/36	Sequencing			RECIST	N3	7
Li	2014	China	167	27–71	87/80	PYRS	FOLFIRI, IRI + beva, IRI+ CAP	180/biweekly	RECIST	N3	7
Gao	2013	China	276	21–79	166/110	Sequencing	FOLFIR, IRI + cetuximab, IRI, XELIRI	180/biweekly	RECIST	N3	7
Okuyama	2011	Japan	52	35–79	32/20	RFLP	FLIRI	100–150/biweekly	RECIST	N3	7
Atasilp	2016	Thailand	44	43–82	26/18	Sequencing	IRI, FOLFIRI + beva, FOLFIRI + cetuximab	180/biweekly, 100/U	RECIST	N4	7
Hazama	2013	Japan	75	U	50/25	Sequencing	FOLFIRI	150/biweekly	RECIST	N3	7
Levesque	2013	Canada	167	61.5	110/57	Sequencing	FOLFIRI, FOLFIRI + vacizumab	180/biweekly	RECIST	N3	7
Bai	2017	China	27	U	U	DFMH	IRI, IRI + cisplatin, IRI+ cisplatin + beva, FOLFIRI, FOLFIRI + beva, IRI + beva, IRI + cisplatin	60/weekly or 130/3 weeks or 50/biweekly or 80/biweekly	RECIST	N3	7

Abbreviations: beva, bevacizumab; CAP, capecitabine; CTC, common terminology criteria; F, female; FLIRI, IRI + 5FU/LV; FOLFIRI, IRI + infusional 5FU+ LV; IFL, 5FU/LV; IROX, IRI +OX; LV, leucovorin; M, male; N, National Cancer Institute Common Toxicity Criteria; PYRS, pyrosequencing; RFLP, reaction-restriction fragment length polymorphism; 5FU, 5-fluorouracil.

### UGT1A1*6 polymorphism and IRI-based chemotherapy TR

Many previous researches analyzed the association between UGT1A1*6 polymorphism and IRI-based chemotherapy TR in different genotypes. According to RECIST medical efficacy appraisal standard, the response rate (RR, RR = CR+PR) and disease control rate (DCR, DCR = CR+PR+SD) were used as end points to evaluate IRI-based chemotherapy TR.

**Figure 2:**
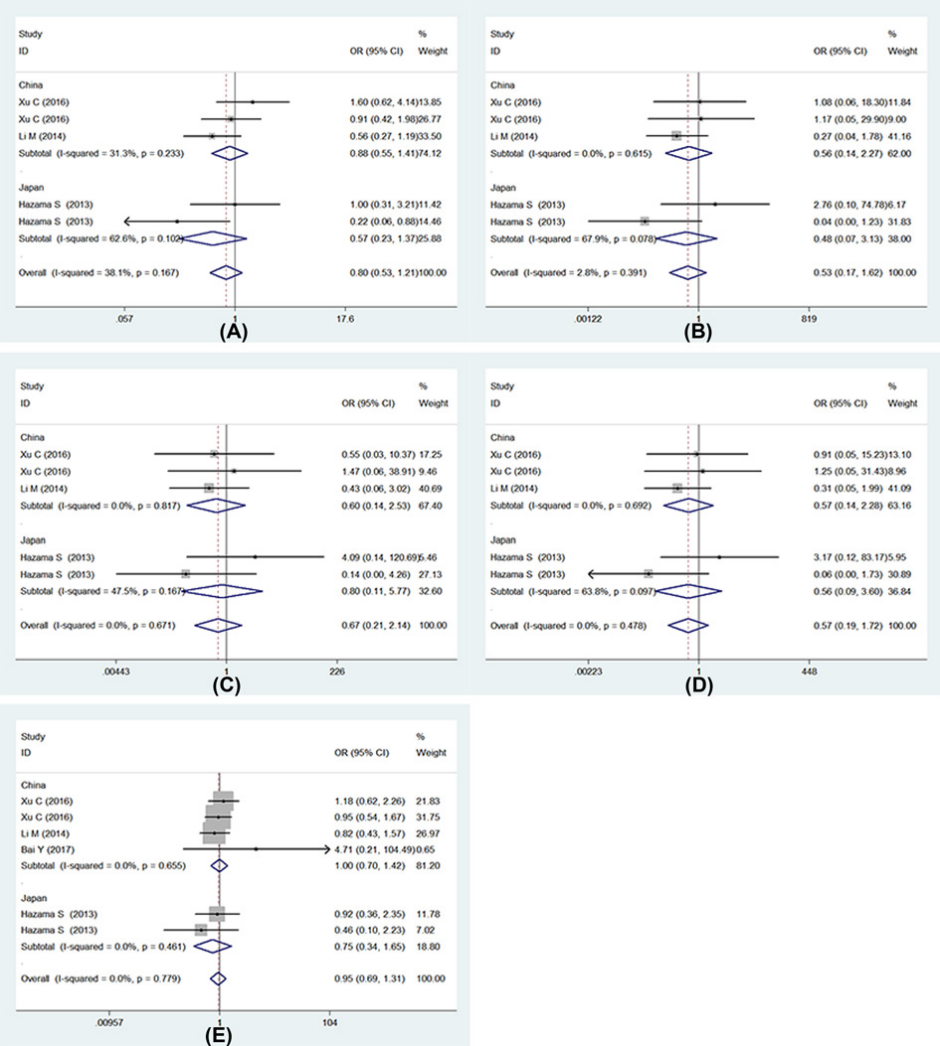
Forests for UGT1A1*6 polymorphism and IRI-based chemotherapy RR (**A**) Represents allele model (G vs. A); (**B**) represents homozygote model (GG vs. AA); (**C**) represents heterozygote model (GA vs. AA); (**D**) represents dominant model (GG+GA vs. AA); (**E**) represents recessive model (GG vs. GA+AA).

Four trails analyzed the RR, and two studies [13,18] described two subgroups RR, respectively. Thus, we decided to evaluate six trails. Five studies listed out the numbers of patients in different genotypes, but one study only listed out the number of wild genotype (GG) and variant genotype (GA+AA). Therefore, five models were used in five studies, and recessive model was used in all trails. As the results show: (1) allele model: (G vs. A: OR = 0.80, 95% CI: 0.53–1.21, *P*=0.29); (2) homozygote model: (GG vs. AA: OR = 0.53, 95% CI: 0.17–1.62, *P*=0.27); (3) heterozygote model: (GA vs. AA: OR = 0.67, 95% CI: 0.21–2.14, *P*=0.50); (4) dominant model: (GG+GA vs. AA: OR = 0.57, 95% CI: 0.19–1.72, *P*=0.32); (5) recessive model: (GG vs. GA+AA: OR = 0.95, 95% CI: 0.69–1.31, *P*=0.76). There was no significant heterogeneity among these models, *I^2^* values were 38.1% (*P*=0.17), 2.8% (*P*=0.39), 0 (*P*=0.67), 0 (*P*=0.48), 0 (*P*=0.78) for allele model, homozygote model, heterozygote model, dominant model and recessive model, respectively. We also performed subgroup analysis by countries, and no associations were observed in different country (Figure 2). Full details are shown in Tables 2 and 3.

**Table 2:** Meta-analysis results for clinical responses and adverse reactions

	G vs. A		GG vs. AA		GA vs. AA		GG+GA vs. AA		GG vs. AA+GA	
	OR (95% CI)	*P*	OR (95% CI)	*P*	OR (95% CI)	*P*	OR (95% CI)	*P*	OR (95% CI)	*P*
**RR**										
**Country**										
**China**	0.88 (0.55, 1.41)	0.60	0.56 (0.14, 2.27)	0.42	0.60 (0.14, 2.53)	0.49	0.57 (0.14, 2.28)	0.43	1.00 (0.70, 1.42)	0.99
**Japan**	0.57 (0.23, 1.37)	0.21	0.48 (0.07, 3.13)	0.44	0.80 (0.11, 5.77)	0.83	0.56 (0.09, 3.60)	0.55	0.75 (0.34, 1.65)	0.47
**Overall**	0.80 (0.53, 1.21)	0.29	0.53 (0.17, 1.62)	0.27	0.67 (0.21, 2.14)	0.50	0.57 (0.19, 1.72)	0.32	0.95 (0.69, 1.31)	0.76
**DCR**										
**Overall**	0.93 (0.59, 1.46)	0.75	1.91 (0.50, 7.28)	0.35	2.29 (0.58, 9.08)	0.24	2.00 (0.53, 7.54)	0.31	0.95 (0.69, 1.31)	0.97
**Diarrhea**										
**Overall**	0.53(0.28, 0.99)	0.05	0.48 (0.23, 0.99)	0.05	0.54 (0.24, 1.23)	0.14	0.49 (0.24, 1.01)	0.06	0.50 (0.24, 1.06)	0.07
Overall*	0.41 (0.28, 0.61)	0.00							0.38 (0.24, 0.60)	0.00
**Neutropenia**										
**Country**										
**China**	0.60 (0.47, 0.76)	0.00	0.29 (0.17, 0.50)	0.00	0.42 (0.24, 0.74)	0.00	0.33 (0.19, 0.55)	0.00	0.42 (0.24, 0.71)	0.00
China*									0.28 (0.18, 0.45)	0.00
**Overall**	0.57 (0.46, 0.71)	0.00	0.28 (0.17, 0.45)	0.00	0.42 (0.26, 0.70)	0.00	0.32 (0.20, 0.52)	0.00	0.40 (0.22, 0.71)	0.00
Overall*									0.35 (0.24, 0.52)	0.00

* represents greater heterogeneity.

**Table 3:** Test for heterogeneity in different analysis

	G vs. A		GG vs. AA		GA vs. AA		GG+GA vs. AA		GG vs. AA+GA	
	*I^2^*	*P*	*I^2^*	*P*	*I^2^*	*P*	*I^2^*	*P*	*I^2^*	*P*
**RR**										
**Country**										
**China**	31.3%	0.23	0.0%	0.62	0.0%	0.82	0.0%	0.69	0.0%	0.66
**Japan**	62.6%	0.10	67.9%	0.08	47.5%	0.17	63.8%	0.10	0.0%	0.46
**Overall**	38.1%	0.17	2.8%	0.39	0.0%	0.67	0.0%	0.48	0.0%	0.78
**DCR**										
**Overall**	0.0%	0.84	0.0%	0.73	0.0%	0.60	0.0%	0.69	0.0%	0.97
**Diarrhea**										
**Overall**	73.9%	0.00	24.2%	0.26	0.0%	0.61	1.2%	0.4	70.5%	0.01
	17.2%	0.31							27.2%	0.24
**Neutropenia**										
**Country**										
**China**	0.0%	0.42	0.0%	0.93	0.0%	0.95	0.0%	0.96	57.9%	0.05
China*									0.0%	0.62
**Overall**	3.1%	0.41	0.0%	0.99	0.0%	0.99	0.0%	1.00	59.2%	0.02
Overall*									46.3%	0.08

* represents greater heterogeneity.

**Figure 3:**
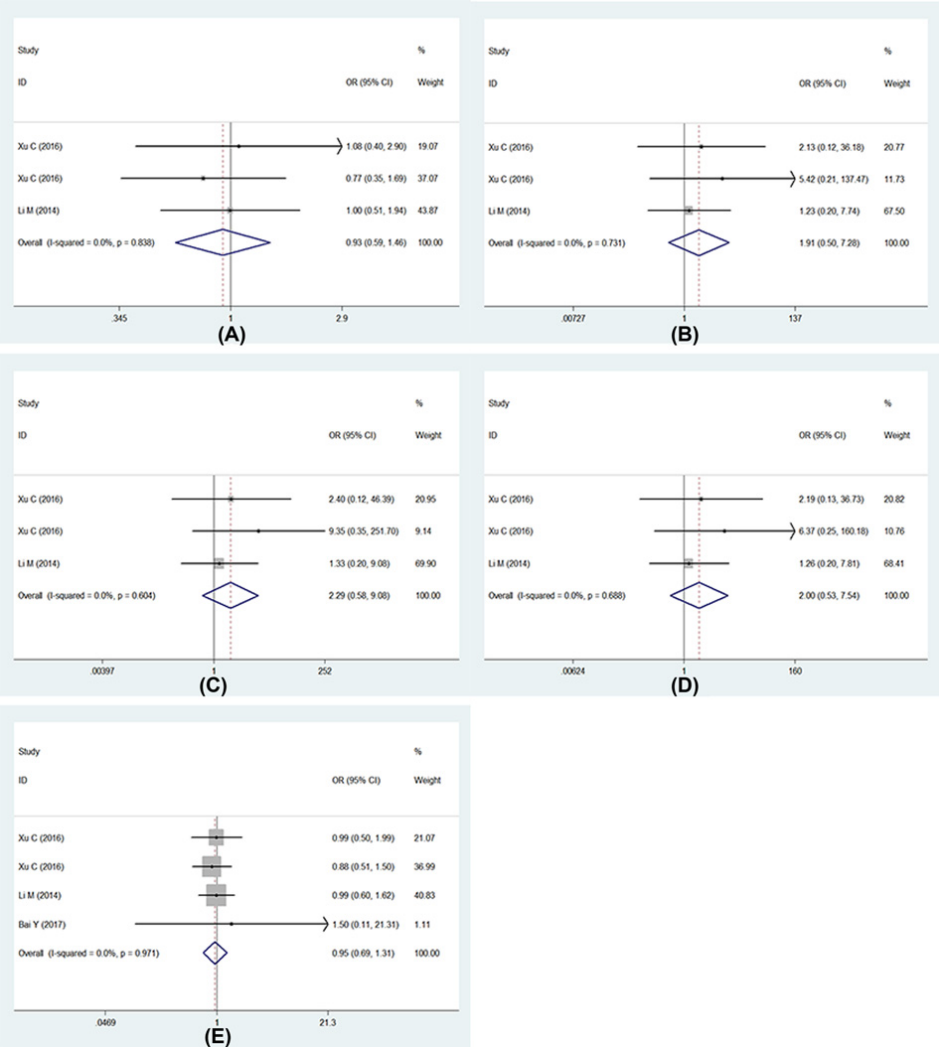
Forests for UGT1A1*6 polymorphism and IRI-based chemotherapy DCR (**A**) Represents allele model (G vs. A); (**B**) represents homozygote model (GG vs. AA); (**C**) represents heterozygote model (GA vs. AA); (**D**) represents dominant model (GG+GA vs. AA); (**E**) represents recessive model (GG vs. GA+AA).

In addition, the DCR was used to evaluate the TR. We analyzed in five models and found no relationship with UGT1A1*6 polymorphism and IRI-based chemotherapy DCR (Figure 3): (1) allele model: (G vs. A: OR = 0.93, 95% CI: 0.59–1.46, *P*=0.75); (2) homozygote model: (GG vs. AA: OR = 1.91, 95% CI: 0.50–7.28, *P*=0.35); (3) heterozygote model: (GA vs. AA: OR = 2.29, 95% CI: 0.58–9.08, *P*=0.24); (4) dominant model: (GG+GA vs. AA: OR = 2.00, 95% CI: 0.53–7.54, *P*=0.31); (5) recessive model: (GG vs. GA+AA: OR = 0.95, 95% CI: 0.69–1.31, *P*=0.97). Full details are shown in Tables 2 and 3.

### UGT1A1*6 polymorphism and IRI-induced severe late-onset diarrhea

**Figure 4.1:**
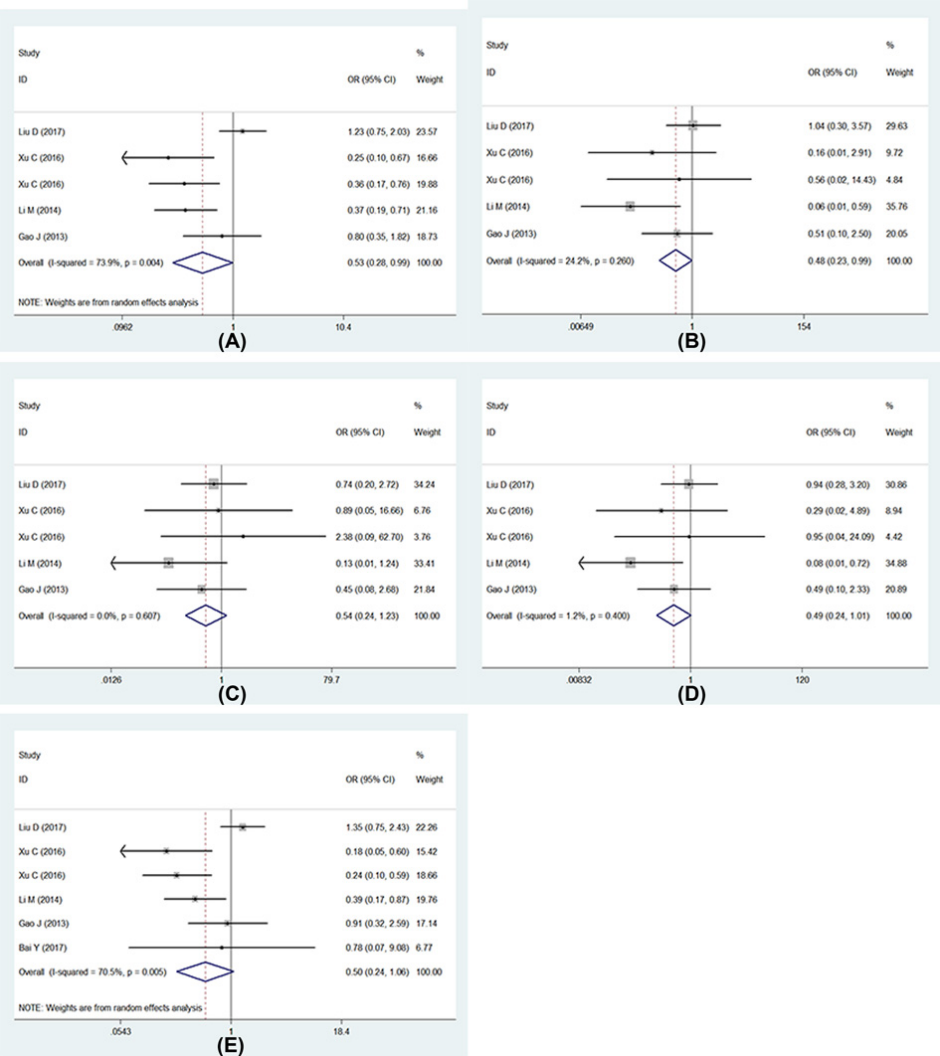
Forests for UGT1A1*6 polymorphism and IRI-induced severe late-onset diarrhea (**A**) Represents allele model (G vs. A); (**B**) represents homozygote model (GG vs. AA); (**C**) represents heterozygote model (GA vs. AA); (**D**) represents dominant model (GG+GA vs. AA); (**E**) represents recessive model (GG vs. GA+AA).

**Figure 4.2:**
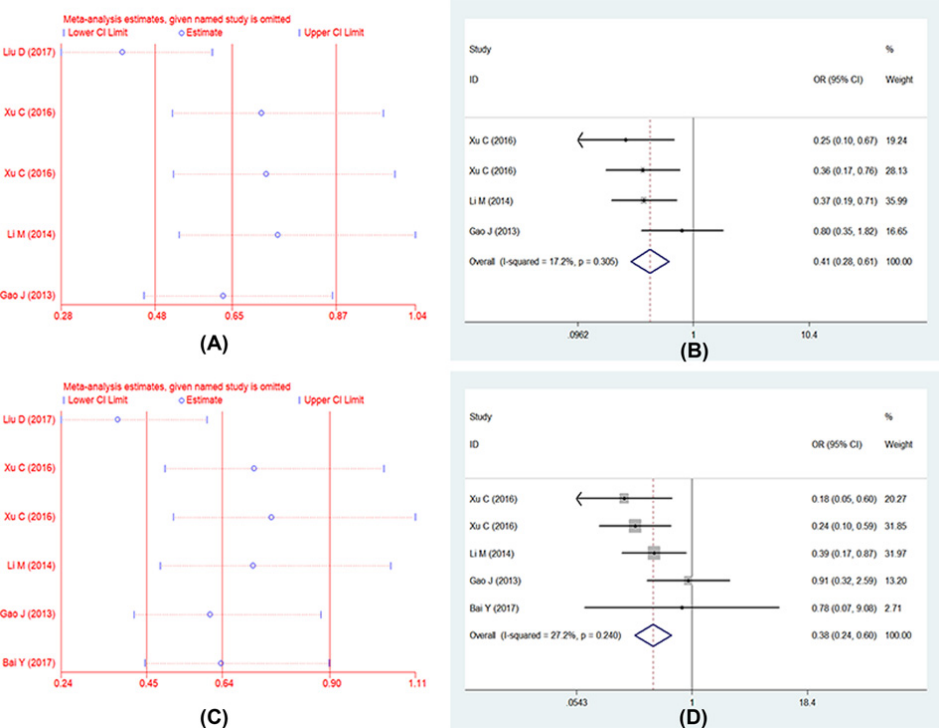
Forests for UGT1A1*6 polymorphism and IRI-induced severe late-onset diarrhea (**A**) Represents sensitive analysis in allele model; (**B**) represents the results of removing heterogeneity in allele model; (**C**) represents sensitive analysis in recessive model; (**D**) represents the results of removing heterogeneity in recessive model.

Five studies described the association between UGT1A1*6 polymorphism and severe late-onset diarrhea, respectively, and one study researched two different nations including Han and Xinjiang Uygur nationalities [13], therefore, six researches were analyzed finally. Whereas one study only listed the number of wild genotype (GG) and variant genotype (GA+AA), we used five models in five studies, and six studies were analyzed in recessive model. As the results show (Figure 4.1): (1) allele model: (G vs. A: OR = 0.53, 95% CI: 0.28–0.99, *P*=0.05); (2) homozygote model: (GG vs. AA: OR = 0.48, 95% CI: 0.23–0.99, *P*=0.05); (3) heterozygote model: (GA vs. AA: OR = 0.54, 95% CI: 0.24–1.23, *P*=0.14); (4) dominant model: (GG+GA vs. AA: OR = 0.49, 95% CI: 0.24–1.01, *P*=0.06); (5) recessive model: (GG vs. GA+AA: OR = 0.50, 95% CI: 0.24–1.06, *P*=0.07). We found the heterogeneity in allele model and recessive model, *I^2^* values were 73.9% (*P*=0.00), 70.5% (*P*=0.01) for allele model and recessive model respectively, the sensitive analysis was performed (Figure 4.2); we found one study had obvious heterogeneity [12], we removed it and analyzed again. Then the heterogeneity decreased and used fixed-effects model, the results showed that UGT1A1*6 polymorphism was associated with late-onset diarrhea (Figure 4.2): (1) allele model (G vs. A: OR = 0.41, 95% CI: 0.28–0.61, *P*=0.00); (2) recessive model (GG vs. GA+AA: OR = 0.38, 95% CI: 0.24–0.60, *P*=0.00). Full details are shown in Tables 2 and 3.

### UGT1A1*6 polymorphism and IRI-induced severe neutropenia

**Figure 5.1:**
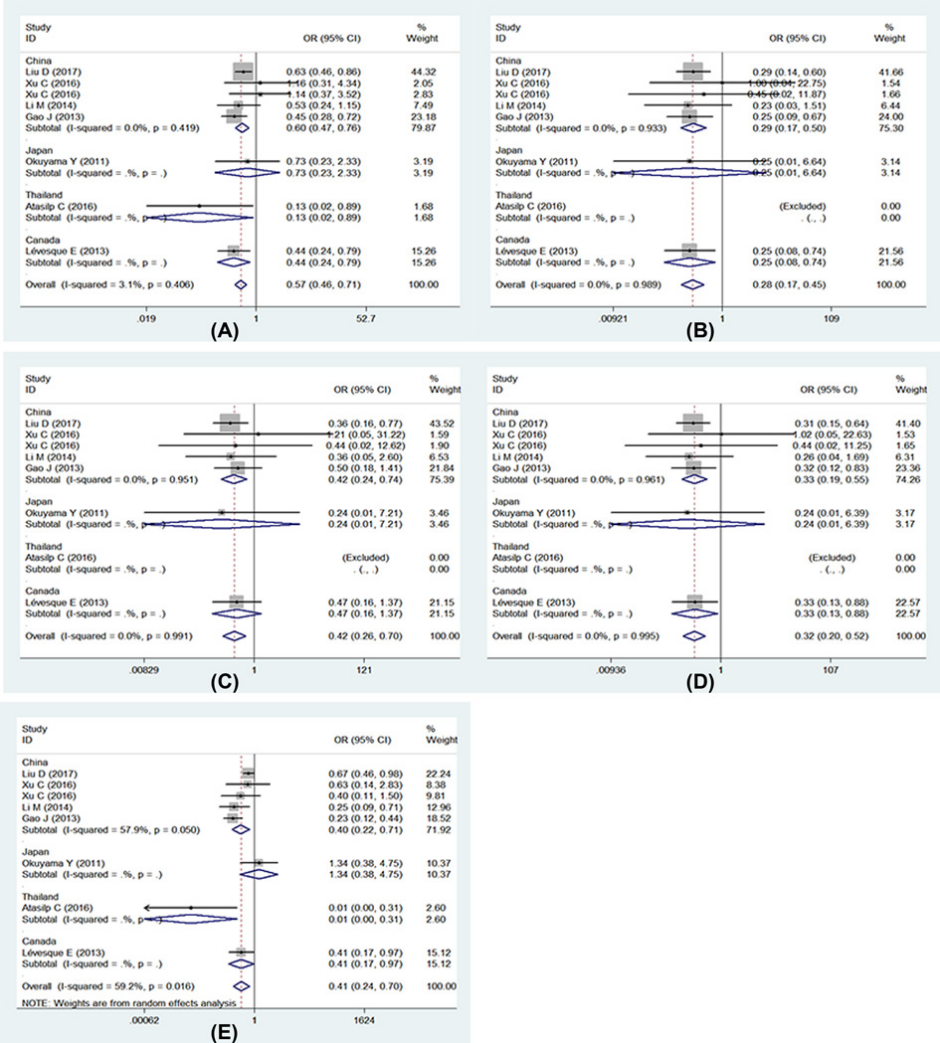
Forests for UGT1A1*6 polymorphism and IRI-induced severe neutropenia (**A**) Represents allele model (G vs. A); (**B**) represents homozygote model (GG vs. AA); (**C**) represents heterozygote model (GA vs. AA); (**D**) represents dominant model (GG+GA vs. AA); (**E**) represents recessive model (GG vs. GA+AA).

**Figure 5.2:**
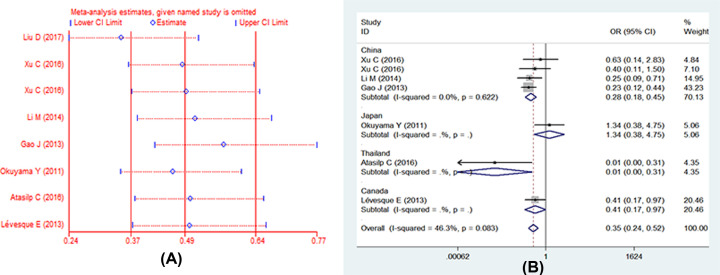
Forests for UGT1A1*6 polymorphism and IRI-induced severe neutropenia (**A**) Represents sensitive analysis in recessive model; (**B**) represents the results of removing heterogeneity in recessive model.

Seven studies described the association between UGT1A1*6 polymorphism and severe neutropenia, respectively, and one study researched two different nations including Han and Xinjiang Uygur nationalities [13], thus eight researches were analyzed finally. Whereas one study only listed the number of wild genotype (GG) and variant genotype (GA+AA), thus four models including allele model (G vs. A), homozygote model (GG vs. AA), heterozygote model (GA vs. AA), dominant model (GG+GA vs. AA) were used in seven studies, and the recessive model analyzed eight studies. No significant heterogeneity was found in all gene models except for recessive model. In recessive model, the random-effects model was used to analyze, and the result showed that UGT1A1*6 polymorphism was the risk of IRI-induced severe neutropenia (GG vs. AA+GA: OR = 0.40, 95% CI: 0.22–0.71, *P*=0.00). Other gene models, the fixed-effects model was used to evaluate. Our results showed that UGT1A1*6 polymorphism was associated with IRI-induced severe neutropenia (Figure 5.1): (1) allele model: (G vs. A: OR = 0.57, 95% CI: 0.46–0.71, *P*=0.00); (2) homozygote model: (GG vs. AA: OR = 0.28, 95% CI: 0.17–0.45, *P*=0.00); (3) heterozygote model: (GA vs. AA: OR = 0.42, 95% CI: 0.26–0.70, *P*=0.00); (4) dominant model: (GG+GA vs. AA: OR = 0.32, 95% CI: 0.20–0.52, *P*=0.00). We further performed sensitive analysis in recessive model (Figure 5.2), and we found one study had obvious heterogeneity [12], we removed it and analyzed again, the heterogeneity decreased statistically. The fixed-effects model was used to analyze again, no obvious change was found (Figure 5.2) (GG vs. GA+AA: OR = 0.35, 95% CI: 0.24–0.52, *P*=0.00) than previous result. Full details are shown in Tables 2 and 3.

Moreover, we then performed the subgroup analyses by different countries. Whereas we only analyzed five models in China, the other countries cannot be analyzed for few studies. There was a statistically increased severe neutropenia in the comparison of five models in China. In different models present different results, we found the significant association in all genetic models between UGT1A1*6 polymorphism and IRI-induced neutropenia in China: (1) allele model: (G vs. A: OR = 0.60, 95% CI: 0.47–0.76, *P*=0.00); (2) homozygote model: (GG vs. AA: OR = 0.29, 95% CI: 0.17–0.50, *P*=0.00); (3) heterozygote model: (GA vs. AA: OR = 0.42, 95% CI: 0.24–0.74, *P*=0.00); (4) dominant model: (GG+GA vs. AA: OR = 0.33, 95% CI: 0.19–0.55, *P*=0.00); (5) recessive model (GG vs. AA+GA:OR = 0.40, 95% CI: 0.22–0.71, *P*=0.00). In recessive model, we also found the significant heterogeneity, so we further performed sensitivity analysis. We found one study had obvious heterogeneity [12], we removed it and analyzed again, the heterogeneity decreased statistically. The fixed-effects model was used to analysis again, the result is the same as before (GG vs. GA+AA: OR = 0.28, 95% CI: 0.18–0.45, *P*=0.00). Full details are shown in Tables 2 and 3.

### Publication bias

**Figure 6:**
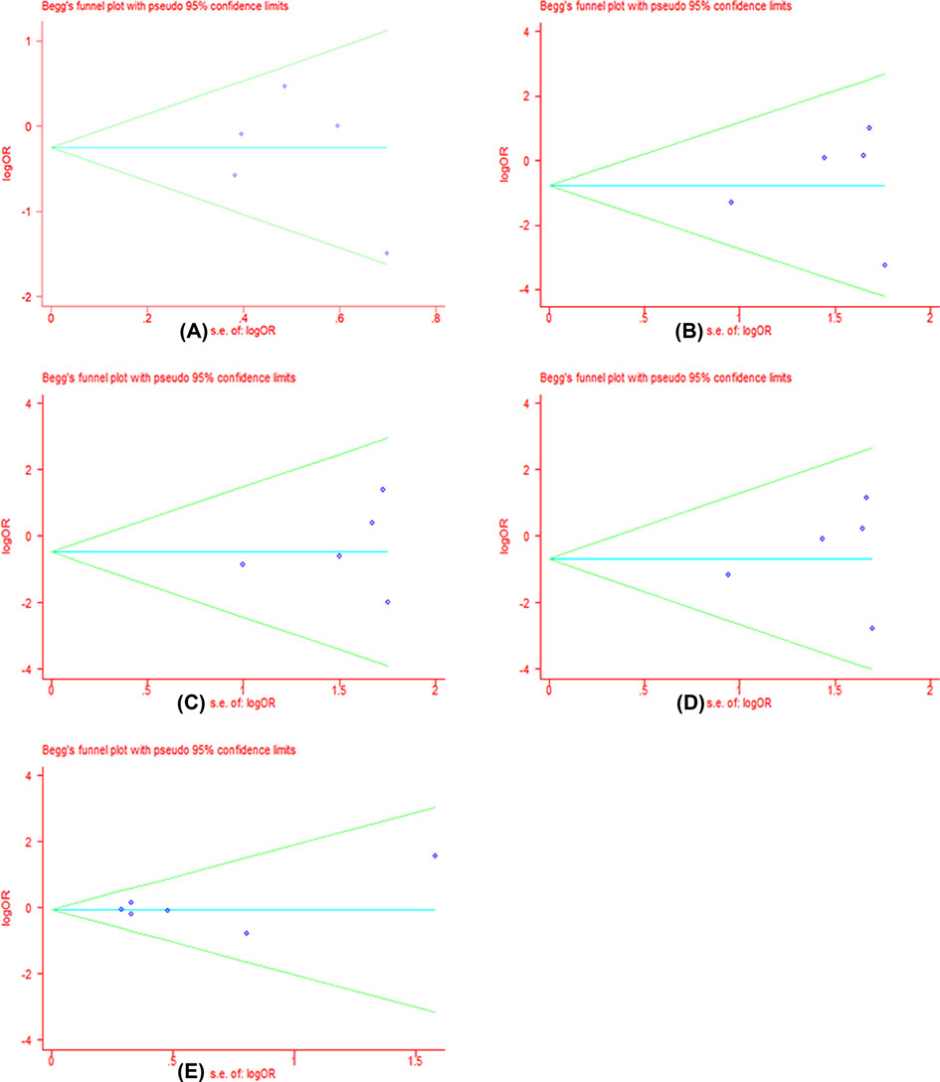
Forests for Begg’s test for RR (**A**) Represents allele model (G vs. A); (**B**) represents homozygote model (GG vs. AA); (**C**) represents heterozygote model (GA vs. AA); (**D**) represents dominant model (GG+GA vs. AA); (**E**) represents recessive model (GG vs. GA+AA).

**Figure 7:**
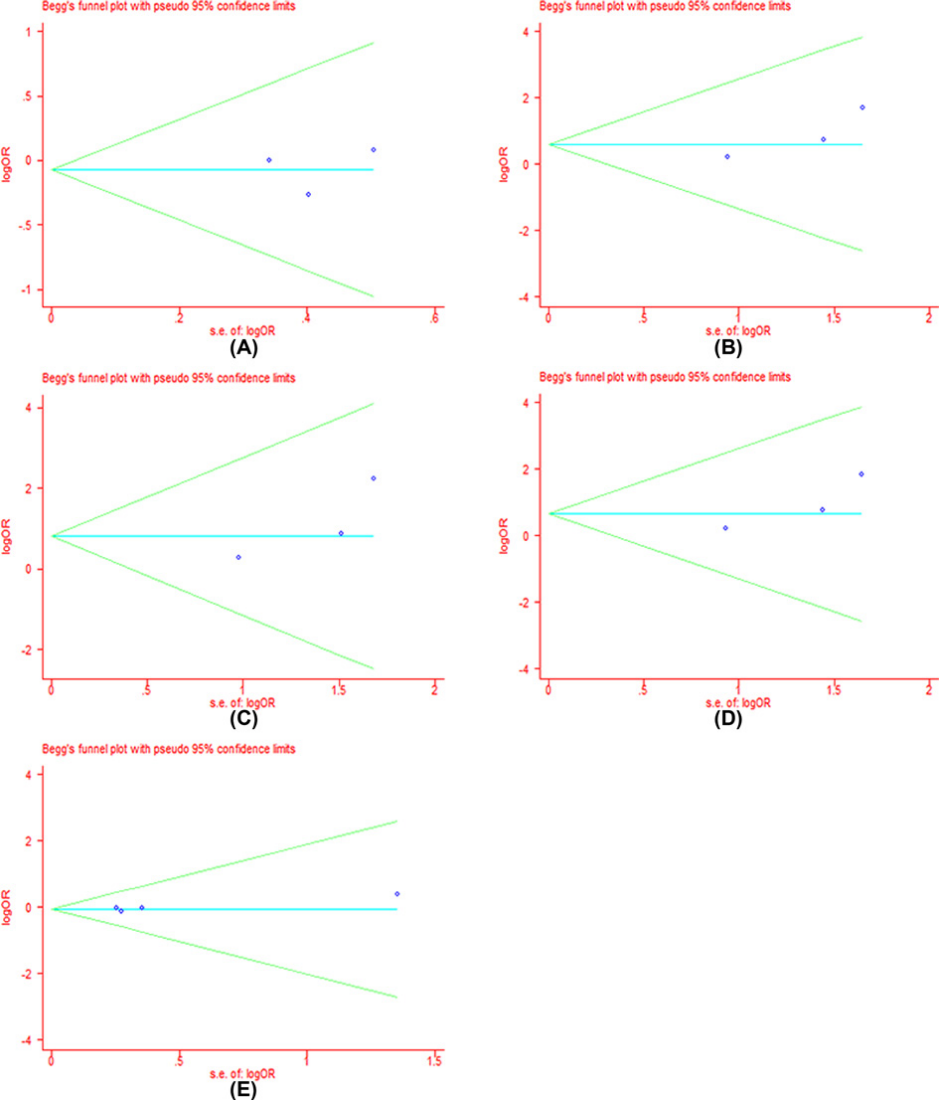
Forests for Begg’s test for DCR (**A**) Represents allele model (G vs. A); (**B**) represents homozygote model (GG vs. AA); (**C**) represents heterozygote model (GA vs. AA); (**D**) represents dominant model (GG+GA vs. AA); (**E**) represents recessive model (GG vs. GA+AA).

**Figure 8:**
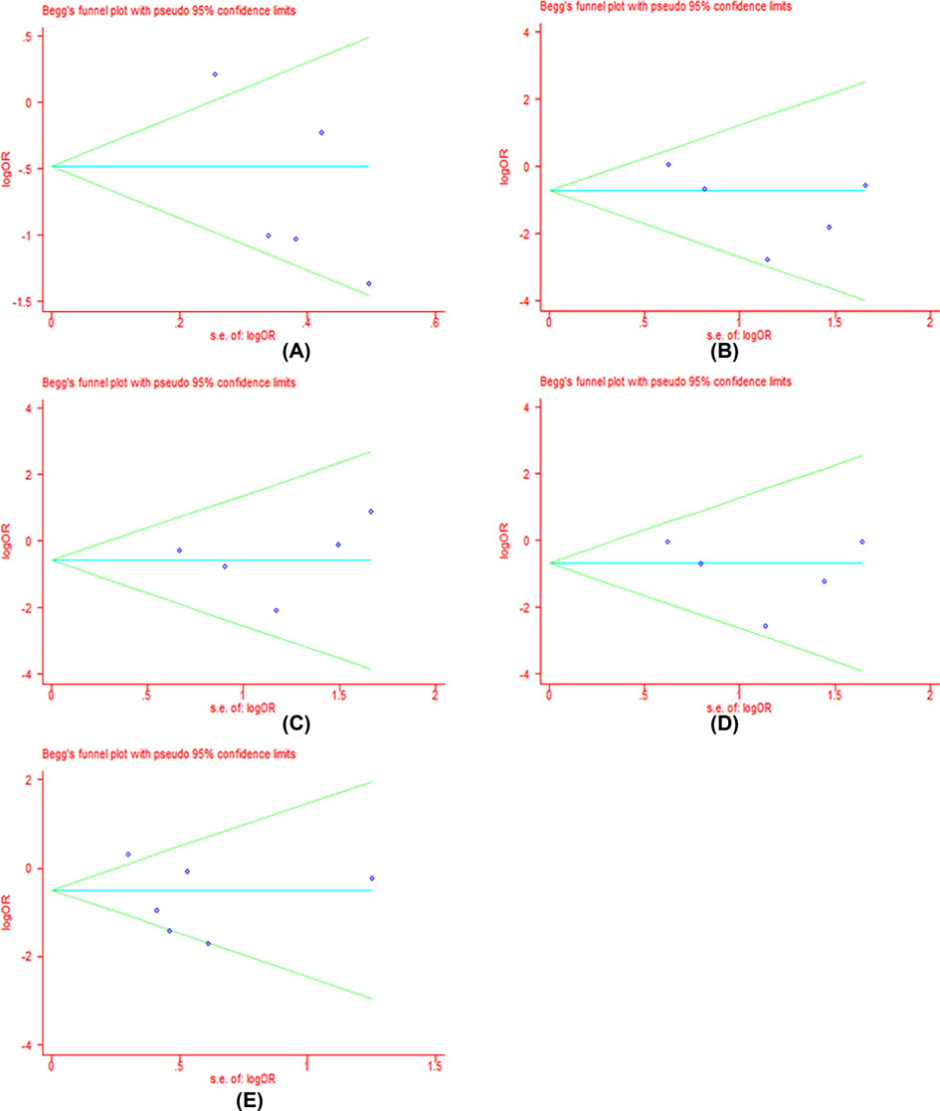
Forests for Begg’s test for IRI-induced severe late-onset diarrhea (**A**) Represents allele model (G vs. A); (**B**) represents homozygote model (GG vs. AA); (**C**) represents heterozygote model (GA vs. AA); (**D**) represents dominant model (GG+GA vs. AA); (**E**) represents recessive model (GG vs. GA+AA).

**Figure 9:**
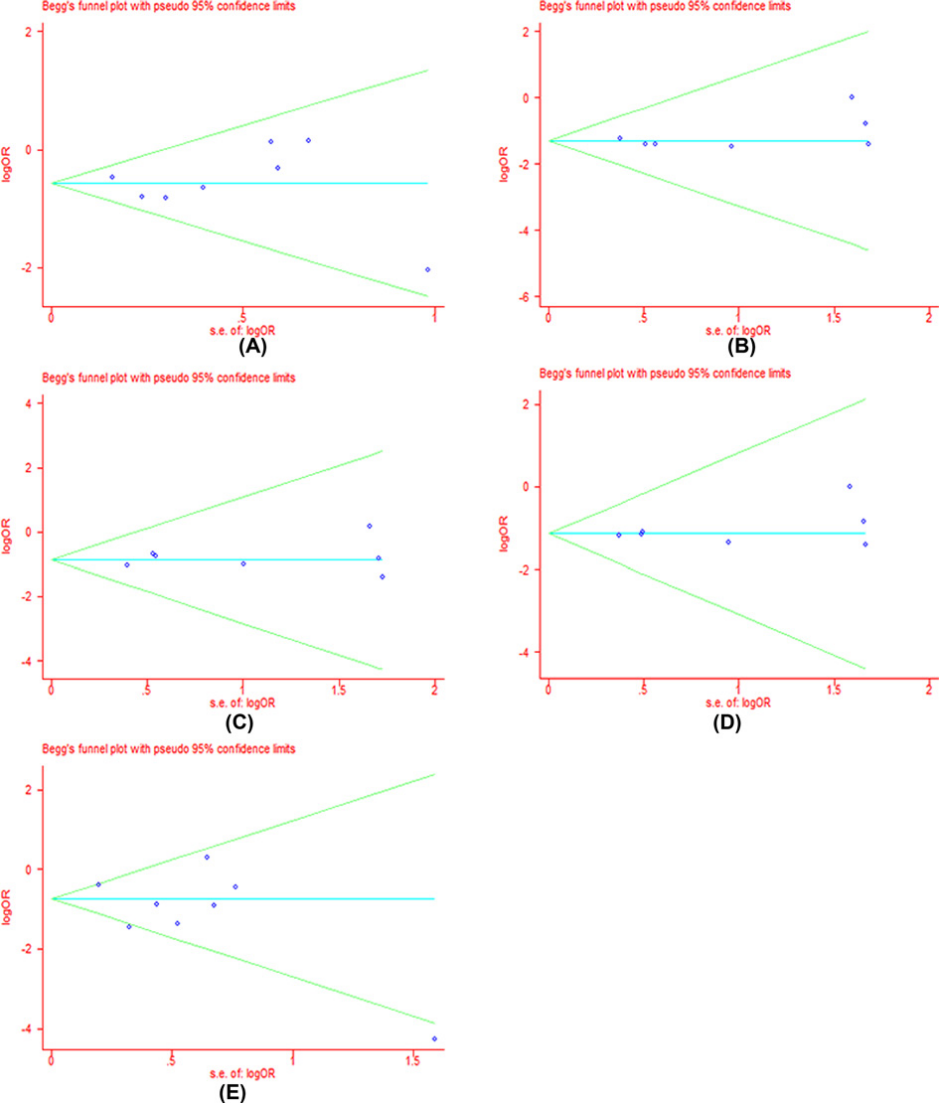
Forests for Begg’s test for IRI-induced severe neutropenia (**A**) Represents allele model (G vs. A); (**B**) represents homozygote model (GG vs. AA); (**C**) represents heterozygote model (GA vs. AA); (**D**) represents dominant model (GG+GA vs. AA); (**E**) represents recessive model (GG vs. GA+AA).

We performed the funnel plot and Begg’s test to assess the publication bias in all included literatures. Publication bias was not found among the studies by funnel plot. Begg’s regression test suggested that there were no obvious statistical publication bias. Details are shown in Table 4 and Figures 6–9.

**Table 4:** *P*-values for Begg’s test for clinical responses and adverse reactions

	G vs. A	GG vs. AA	GA vs. AA	GG+GA vs. AA	GG vs. AA+GA
	*P*	*P*	*P*	*P*	*P*
**RR**	0.81	1.00	0.81	0.81	1.00
**DCR**	1.00	0.30	0.30	0.30	0.73
**Diarrhea**	0.46	0.81	0.81	0.81	0.71
**Neutropenia**	0.90	0.37	1.00	0.76	0.90

## Discussion

Recent advances in chemotherapy for CRC, IRI-based chemotherapy treatment as an effective way for CRC patients and was considered to be the first-line treatment option. However, the obvious concern is efficacy and toxic side effects, especially the serious toxicity restricted its application, such as severe neutropenia and diarrhea. A large of studies found that variations of genes linked with efficacy and toxicity of IRI-based chemotherapy for CRC. UGT1A1 linked with activity of glucuronidation, and involves in the metabolism of IRI. Studies have indicated an association between UDP-glucuronosyltransferase-1A1 (UGT1A1) genetic polymorphisms and IRI-induced toxicity. UGT1A1 gene concludes many SNPs [25,26], and SNPs in candidate gene significantly associated with transcription or translation or regulation [27]. UGT1A1*28 is a member of family in SNPs of *UGT1A1* gene, previous meta-analysis evaluated the impact of UGT1A1*28 polymorphisms with IRI-induced toxicity, and demonstrated UGT1A1*28 polymorphisms may be considered as a marker of IRI-induced toxicity in chemotherapy of cancer [28]. In 2005, the U.S. Food and Drug Administration (FDA) recommended that the UGT1A1*28 was noted as a label for patients [29]. The other SNPs in *UGT1A1* gene, the UGT1A1*6 (rs4148323) polymorphism is a missense polymorphism, which effects the translation of UGT1A1 protein (Gly^71^Arg) [30], the substitution of Gly influence the hydrophobicity and secondary structure of protein and the efficiency of SN38 glucuronidation activity may be decreased [30]. A large number of researches show UGT1A1*6 associated with the efficacy and toxicities of IRI-based chemotherapy in CRC, but the conclusions are still not to be agreed. Previous studies found UGT1A1*6 polymorphism was highly related to RR in Asians [12,13,17]. Xu et al. [13], and reported that wild UGT1A1*6 genotype has significant lower late-onset diarrhea, but no difference in neutropenia between wild genotype and mutant genotype in Xinjiang province of China. However, Gao et al. [15] studied that UGT1A1*6 polymorphism was closely associated with severe neutropenia, but not linked with late-onset diarrhea. Moreover, the study in Thai did not showed that UGT1A1*6 polymorphism significantly increased severe neutropenia [17]. Based on the controversial conclusions, we performed a meta-analysis to comprehensively investigate the association between UGT1A1*6 polymorphism and efficacy and adverse reaction. The present study describes an important molecular biomarker in chemotherapy treatments for CRC, especially in IRI-based combination chemotherapy.

Most studies reported that no association between UGT1A1*6 polymorphism and clinical response among the different genotypes. In this meta-analysis, no relationship was found between UGT1A1*6 polymorphism and clinical response, same results were analyzed in subgroup analysis. Our results are similar to previous studies.

In the present study, we found that UGT1A1*6 polymorphism is a risk variant for severe drug toxicities in IRI-based chemotherapy with CRC patients. Our finding demonstrated that patients carrying base mutation increasingly likely to encounter severe neutropenia (grade III–IV) in all models. In subgroup analysis, UGT1A1*6 polymorphism still increases the risk of severe neutropenia. Similarly, UGT1A1*6 polymorphism increase risk of severe diarrhea (grade III–IV) in allele comparison and homozygote comparison, the results of subgroup analysis is same to overall analysis.

Compared with previous meta-analysis, our study was the first report to estimate the relation between UGT1A1*6 polymorphism and clinical response and toxicity in CRC. The present study analyzed the association between UGT1A1*6 polymorphism and IRI-based chemotherapy TR and toxicity, and found the genotyping of UGT1A1*6 polymorphism may be useful for clinical application. Although we attempt to explore their clinical relevance, several limitations still exist in our research. First, many difference among primary studies, including chemotherapy regimens, research method and doses. Especially, different chemotherapy regimens were used in individual treatment, such as IRI + infusional 5FU+ LV (FOLFIRI), IRI and cisplatin, which would influence the efficacy and adverse reaction. Second, the variability in IRI doses maybe a source of heterogeneity, but no effective data were used to subgroup analyses by IRI doses. Third, the toxicity clinical responses were related to gender, as a study reported the incidence of serve neutropenia was higher in female than male, but no effective data were collected for analysis by gender. In addition, part of studies only included wild genotype and variant genotype, and other models were not analyzed except recessive model. Moreover, the interference of other factors, such as environmental and other genetic factors, as ABCB1 C3435T polymorphism. Finally, the sample size was limited. Thereby, more studies with a larger sample sizes and high quality clinical studies need to research, and enhance the reliability and stability of the meta-analysis.

## Conclusion

In conclusion, this meta-analysis suggested that the UGT1A1*6 polymorphism linked with IRI-induced adverse reaction with CRC, especially increase the incidence of serve late-onset diarrhea and neutropenia. No relationship was found between UGT1A1*6 polymorphism and clinical response.

## Data Availability

All data generated or analyzed during the present study are included in this published article.

## Abbreviations

beva: bevacizumab
CR: complete response
CRC: colorectal cancer
DCR: disease control rate
FOLFIRI: IRI + infusional 5FU+ LV
IRI: irinotecan
NOS: Newcastle–Ottawa scale
OR: odds ratio
PR: partial response
RECIST: Response Evaluation Criteria in Solid Tumors
RR: response rate
SD: stable disease
TR: tumor response
UGT: UDP-glucuronosyltransferase
